# Muscle growth differences in Lijiang pigs revealed by ATAC-seq multi-omics

**DOI:** 10.3389/fvets.2024.1431248

**Published:** 2024-08-26

**Authors:** Yi Lan, Dawei Yan, Xinpeng Li, Chunlu Zhou, Ying Bai, Xinxing Dong

**Affiliations:** ^1^College of Animal Science and Technology, Yunnan Agricultural University, Kunming, China; ^2^School of Life Sciences and Food Engineering, Hebei University of Engineering, Handan, Hebei, China

**Keywords:** ATAC-seq, multi-omics, growth, muscle, Lijiang pigs

## Abstract

As one of the largest tissues in the animal body, skeletal muscle plays a pivotal role in the production and quality of pork. Consequently, it is of paramount importance to investigate the growth and developmental processes of skeletal muscle. Lijiang pigs, which naturally have two subtypes, fast-growing and slow-growing, provide an ideal model for such studies by eliminating breed-related influences. In this study, we selected three fast-growing and three slow-growing 6-month-old Lijiang pigs as subjects. We utilized assay for transposase-accessible chromatin with sequencing (ATAC-seq) combined with genomics, RNA sequencing, and proteomics to screen for differentially expressed genes and transcription factors linked to increased longissimus dorsi muscle volume in Lijiang pigs. We identified 126 genes through ATAC-seq, including *PPARA*, *TNRC6B*, *NEDD1*, and *FKBP5*, that exhibited differential expression patterns during muscle growth. Additionally, we identified 59 transcription factors, including Foxh1, JunB, Mef2 family members (Mef2a/b/c/d), NeuroD1, and TEAD4. By examining open chromatin regions (OCRs) with significant genetic differentiation, genes such as *SAV1*, *CACNA1H*, *PRKCG*, and *FGFR4* were found. Integrating ATAC-seq with transcriptomics and transcriptomics with proteomics, we identified differences in open chromatin regions, transcription, and protein levels of *FKBP5* and *SCARB2* genes in fast-growing and slow-growing Lijiang pigs. Utilizing multi-omics analysis with R packages, we jointed ATAC-seq, transcriptome, and proteome datasets, identifying enriched pathways related to glycogen metabolism and skeletal muscle cell differentiation. We pinpointed genes such as MYF6 and HABP2 that exhibit strong correlations across these diverse data types. This study provides a multi-faceted understanding of the molecular mechanisms that lead to differences in pig muscle fiber growth.

## 1 Introduction

In pig production, the growth and hypertrophy of skeletal muscle directly influence the production output and quality of pork. Pork production is a complex regulatory process greatly affected by factors such as breed, age, and nutrition. Understanding the differences in this process is challenging owing to the complex multi-level regulatory mechanisms involved. In eukaryotic cells, DNA replication involves the unwinding of DNA sequences tightly wound with nucleosomes in the chromatin, forming open chromatin regions (OCRs) (1, 2). OCRs are prone to interactions required for DNA replication and transcription, including those with transcription factors, histones, and other proteins that bind to the nucleosome in OCRs. Competition between these interactions alters gene expression (3). Despite only constituting 2–3% of the genome, OCRs account for more than 90% of transcription factor binding sites (4).

RNA sequencing (RNA-seq) enables the identification of candidate genes linked to variances in porcine muscle growth through the analysis of gene expression levels, which represent outcomes of post-transcriptional processes. To comprehensively understand the regulatory mechanisms involved in epigenetic and transcriptional processes, it is essential to integrate genomic, assay for transposase-accessible chromatin with sequencing (ATAC-seq), RNA-seq, and proteomics data. ATAC-seq plays a crucial role in the study of chromatin accessibility, revealing accessible regions of the genome and helping to predict transcription factor binding sites, thereby linking the control of gene transcription to gene expression. Therefore, conducting a multi-omics analysis to identify the key genes and transcription factors responsible for these disparities will provide valuable insights into the development and growth of porcine skeletal muscle.

Previous studies have utilized similar multi-omics tools to investigate skeletal muscle development and growth in pigs. They have identified transcription factors related to skeletal muscle development, such as MEF2C, CEBP, TFAP4, MAX, NHLH1, MYOD1, SP1, EGR1, and PVALB, using a combination of RNA-seq and ATAC-seq techniques. Additionally, genes including *THRSP*, *ASNS*, *CARNS1*, *G0S2*, *ACBD7*, and *TMEM220* were identified (5–8). It was demonstrated through small interfering RNA and overexpression experiments that ACBD7 could promote the proliferation of porcine skeletal muscle cells. Using ATAC-seq and RNA-seq data from 20 tissues (including muscle) of 6-month-old sows, Jiang et al. found a more conserved pattern of regulation by regulatory elements proximal to the transcription start site (TSS), whereas distal regulatory elements were more tissue-specific (9). Furthermore, Cai et al. comprehensively analyzed the myogenic differentiation of embryonic pigs using a single-cell transcriptome and single-cell ATAC-seq; constructed the individual differentiation trajectory of porcine skeletal muscle; and identified two regulatory factors, EGR1 and RHOB, that are crucial for the development of porcine embryonic muscle fibers (10). Zhao et al. combined ATAC-seq, ChIP-seq and RNA-seq data to screen for differentially expressed genes (DEGs) and proteins and identified *IGF2*, *IYD*, *MLC1*, *MYH7B*, and *PDK4*, and transcription factors such as the Mef2 family, MyoD, Myf5, and MyoG (11). Xu systematically integrated RNA-seq, ATAC-seq, ChIP-seq and Hi-C techniques to comprehensively determine cis-regulatory elements in 12 tissues, including skeletal muscle, from four different pig breeds (12). Hu et al. combined microRNAomic data with previously published datasets from multiple histologies, including ChIP-seq and ATAC-seq data, and identified 19 motifs, including MYOG, MYF5, MEF2A, and SIX1, that were significantly enriched in muscle tissues (13). However, the mechanisms underlying differences in muscle development and growth among pigs with different growth rates and body types remain unclear.

The Lijiang pig is a native pig breed of Yunnan Province, China. Lijiang pigs have excellent meat quality and can adapt to plateau environments. They can be categorized into two groups: fast-growing Lijiang pigs, which are larger, and slow-growing Lijiang pigs, which are smaller. Research conducted by the Lijiang pig conservation group at Lijiang Yaoyuan Farm Co., Ltd. in Lijiang City, Yunnan Province found that 8-month-old fast-growing Lijiang pigs had an average weight of 96.23 ± 4.33 kg and an average daily gain (ADG) of 604 ± 32 g during the fattening period. In contrast, slow-growing Lijiang pigs weighed 60.72 ± 3.98 kg with an ADG of 331 ± 49 g, these findings highlight significant differences in weight and growth rate between the two types of Lijiang pigs (14). However, the molecular mechanisms underlying these differences remain unclear. Studying muscle growth rates within the same breed under similar age and nutritional conditions can help eliminate genetic background differences that occur when studying different breeds. This approach can effectively identify the key genes that influence muscle growth. Liu et al. conducted a similar study on different body sizes of Meishan pigs and that *NR6A1* missense mutations and *RSAD2-CMPK2* and *COL3A1* haplotypes caused body size differences between medium and mini Meishan pigs (15).

In this study, we employed ATAC-seq to quantify chromatin accessibility in the longissimus dorsi muscles of 6-month-old three fast and three slow-growing Lijiang pigs by mapping the chromatin landscape at these two growth rates. We predicted the transcription factors associated with muscle growth and annotated differential genes within differentially expressed peaks. By integrating ATAC-seq, genomics, RNA-seq, and proteomics data, we elucidated the association between chromatin accessibility and variations in gene expression levels. This comprehensive approach also allowed us to identify potential transcription factors that contribute to the differences in muscle growth rates observed in Lijiang pigs. Using multi-omics analysis, we investigated muscle growth in Lijiang pigs, offering significant insights into the molecular mechanisms associated with muscle growth in indigenous Chinese pig breeds, and providing a basis for cultivating excellent high-yielding pig breeds.

## 2 Experimental materials and methods

### 2.1 Ethical statement

All experimental procedures were approved by the Animal Welfare Committee of the Yunnan Agricultural University (202303057).

### 2.2 Sample collection and sequencing

Three fast-growing Lijiang pigs (LJF) and three slow-growing Lijiang pigs (LJS) with similar birth dates were selected for this study (Supplementary Table S1). The two groups of pigs were born on similar dates and belonged to different full-sibling families within the Lijiang pig breed. They were kept under uniform conditions with identical diets and had unrestricted access to feed. All pigs were slaughtered at the age of 6 months, and fasted overnight. After slaughter, the longissimus dorsi muscle tissue was promptly collected, immediately preserved in liquid nitrogen, and then stored in a refrigerator set at −80°C. Transposition was performed in accordance with the protocols of ATAC-Seq Assay Kits (Active Motif, Carlsbad, CA, USA). Purification was conducted using a MinElute PCR Purification Kit (Qiagen, Hilden, Germany) and amplification was performed using a Nextera DNA Library Preparation Kit (Illumina, USA). Library quality was evaluated using an Agilent 2,100 Bioanalyzer (Agilent, Santa Clara, CA, USA). Paired-end sequencing was performed on an Illumina NovaSeq 6000 platform, with a read length of 150 bp.

The RNA-seq (PRJNA1018447) and proteomic (PXD045863) data sets were collected from the same batch of pigs as the ATAC-seq data from previous experiments conducted by our research group (16). Genomic data (PRJNA942216) were obtained for 44 unrelated Lijiang pigs (17).

### 2.3 Analysis of ATAC data

ATAC data were subjected to quality control using Fastp software (v0.23.2) to assess library quality, remove adapter sequences, and filter out low-quality reads (−-detect_adapter_for_pe –D –M 15 –q15 –u 40 –length_required 30) (18). Subsequently, the data were aligned to the pig reference genome (*Sus scrofa* 11.1) using BWA (v0.7.17-r1188) software (19). Y chromosome and mitochondrial sequences were excluded using SAMtools (v1.6) (20). Coordinate transformation and insertion fragment counting were performed using ATACseqQC (v1.24.0) (21). Peak calling was conducted using MACS2 (v2.2.8) to identify open chromatin regions (−f BAMPE -nomodel -shift −100 --extsize 200 -q 0.05) (22). Tissue-specific peaks were merged into a standardized peak format using BEDTools (v2.25.0) and stored in a bed file (23). The narrow peaks underwent annotation analysis using the HOMER annotatePeaks.pl. script (v4.11), while the bamCoverage function of deepTools was used to generate bigwig files for peak visualization in IGV software (v2.16.1) (24–26). The ComputeMatrix function was used to analyze the read distribution within 2,000 bp of the transcription start sites (TSS), with plotProfile function was used to draw signal density distribution maps. Principal component analysis was performed using all features from the ATAC-seq data. Differential OCRs between fast-growing and slow-growing individuals were analyzed using the DiffBind R package (v3.10.1), where peaks with FDR < 0.05 indicated significant differences (27). The findMotifsGenome.pl script in HOMER was applied with default parameters to search for motifs within the differential peaks (*p* < 0.05). Enrichment analysis using Gene Ontology (GO) and Kyoto Encyclopedia of Genes and Genomes (KEGG) were conducted for genes associated with significant differential peaks.

### 2.4 Genomic data analysis

The quality control and comparison of genome resequencing data were consistent with those reported by Yang et al. (17). In order to obtain a window of high genetic differentiation among fast-growing and slow-growing Lijiang pigs, we used VCFtools (v0.1.16) (28) to calculate the genetic differentiation index (Fst) and nucleotide diversity (*π*) of the SNP data, and set the window to 20 kb and step size to 10 kb. According to the top 5% of Fst value and the bottom 5% of *π* value, the window is considered to have significant genetic differentiation and small genetic variation. The intersectBed function of BEDTools software was then employed to overlap the tissue peak of ATAC-seq with the identified genomic regions of interest. The overlapping regions were further analyzed for gene annotation. Gene functions were examined through enrichment analysis based on Gene Ontology (GO) and Kyoto Encyclopedia of Genes and Genomes (KEGG).

### 2.5 Transcriptome and proteome data analysis

Raw data filtering and comparisons of the transcriptome and proteome were consistent with those reported by Liu et al. (16). The DEseq2 package (v1.40.2) (29) in R language was used to perform differential expression analysis on RNA-seq data from fast and slow-growing Lijiang pigs, resulting in the identification of DEGs based on the following cut-off values: |log_2_(fold change)| > 1 and adjusted *p* < 0.05. Differential analysis of the detected proteins was performed using the R software package DEP (v1.22.0) (30), and differentially expressed proteins were identified based on the following criteria: *p* < 0.05 and |log_2_(fold change)| > 1.5, After annotation, combined analysis was conducted with the DEGs identified using RNA-seq. Gene functions were assessed via enrichment analyses using GO and KEGG databases.

### 2.6 Joint analysis of ATAC-seq, transcriptome, and proteome data

Differential analysis of gene sets filtered from ATAC-seq, transcriptome, and proteome data was performed separately, followed by Gene Ontology (GO) enrichment analysis. Integration of these gene sets was conducted using the ActivePathways package in R, employing Fisher’s method for combining *p*-values and Bonferroni correction for multiple testing, with a significance threshold set at adjusted *p* < 0.05 (31). Subsequently, the expression matrices of these gene sets were subjected to multi-block sparse partial least squares discriminant analysis (block-sPLS-DA) using the “block.splsda” function from the mixOmics R package (32, 33). This analysis aimed to identify the most important features in each multiomic data that discriminate the two pig lines, LJF and LJS. First principal component of the identified features from each multiomic dataset were visualized using the plotDiablo function. The circosPlot function was utilized to generate a circular plot based on a predefined correlation threshold of 0.9.

## 3 Results

### 3.1 Sample overview

**Figure 1:**
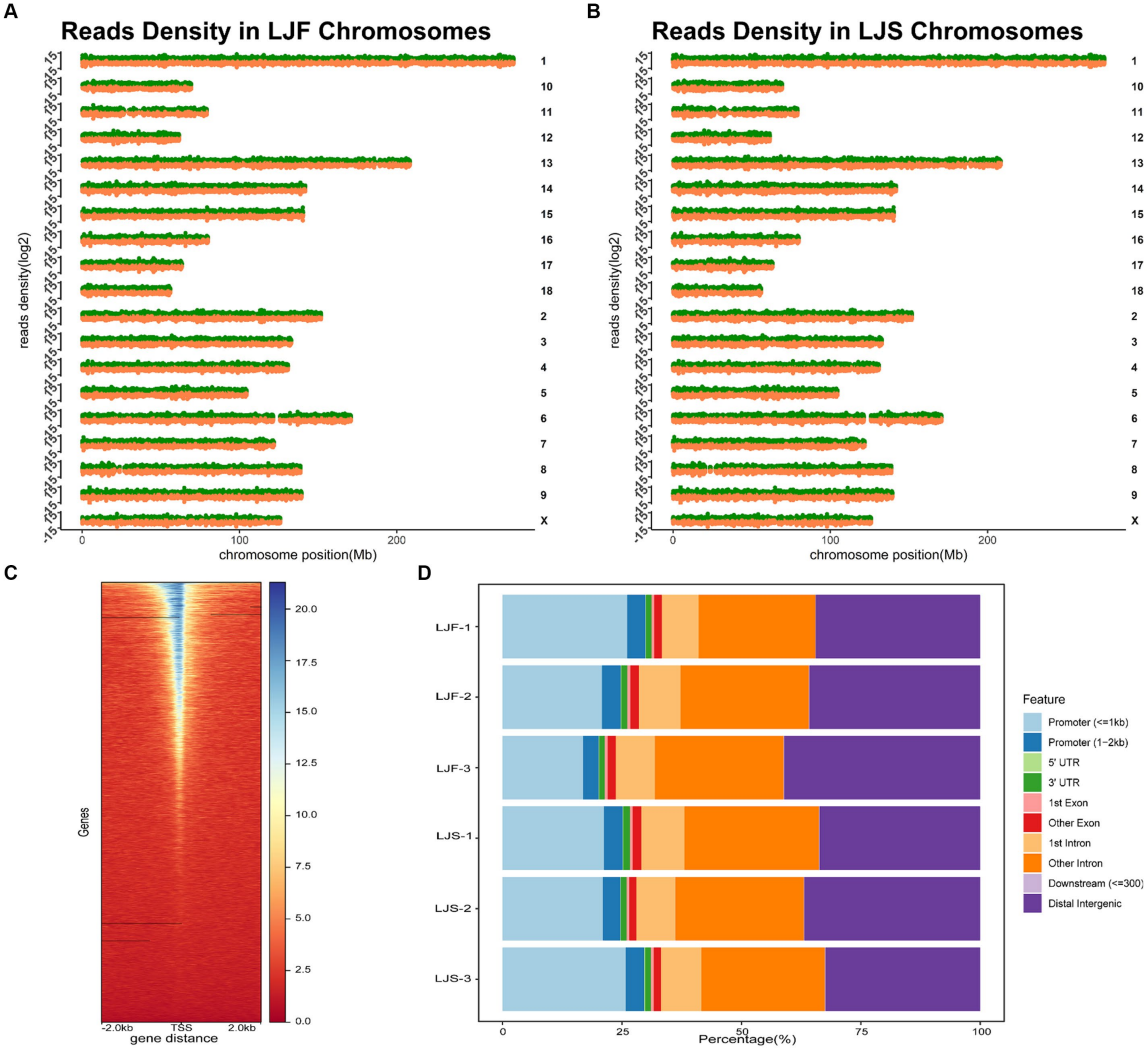
Overall information regarding ATAC-seq samples. Chromosomal distribution of peaks in the **(A)** fast-growing Lijiang (LJF)-1 and **(B)** slow-growing Lijiang (LJS)-1 pig samples. **(C)** Enrichment analysis of assay for transposase-accessible chromatin with sequencing (ATAC-seq) signals within 2 kb around the transcription start site was performed for the LJF-1 sample. **(D)** Annotation results were obtained for open chromatin regions (OCRs) identified in the six samples.

We conducted ATAC-seq analysis of the longest dorsal muscles of Lijiang pigs and integrated genomic, RNA-seq, and proteomic data to elucidate the growth disparities between the muscles of slow and fast-growing Lijiang pigs. Sample and quality control information for the genomic, RNA-seq, and proteomic data are provided in Supplementary Table S1. ATAC-seq generated a total of 428,961,569 clean reads (Supplementary Table S2), with a clean read rate of 92.30 ± 5.04%. The fragment length distributions of the libraries matched the expected pattern (Supplementary Figure S3). Analysis of the peak distribution on the chromosomes revealed comprehensive coverage across most regions of each chromosome (Figures 1A,B). The most identifiable accessible regions were enriched at TSSs within a range of 2 kb, indicating the involvement of OCRs in transcriptional regulation (Figure 1C). The whole-genome functional annotation included intronic regions, intergenic regions, promoters, exons, 5’UTRs and 3’UTRs to annotate all OCRs. On average, approximately 73,040 highly confident OCRs were identified, with the majority being annotated to non-coding regions, such as intronic and intergenic regions, as well as promoter regions (Figure 1D).

### 3.2 Clustering of samples and differential analysis

**Figure 2:**
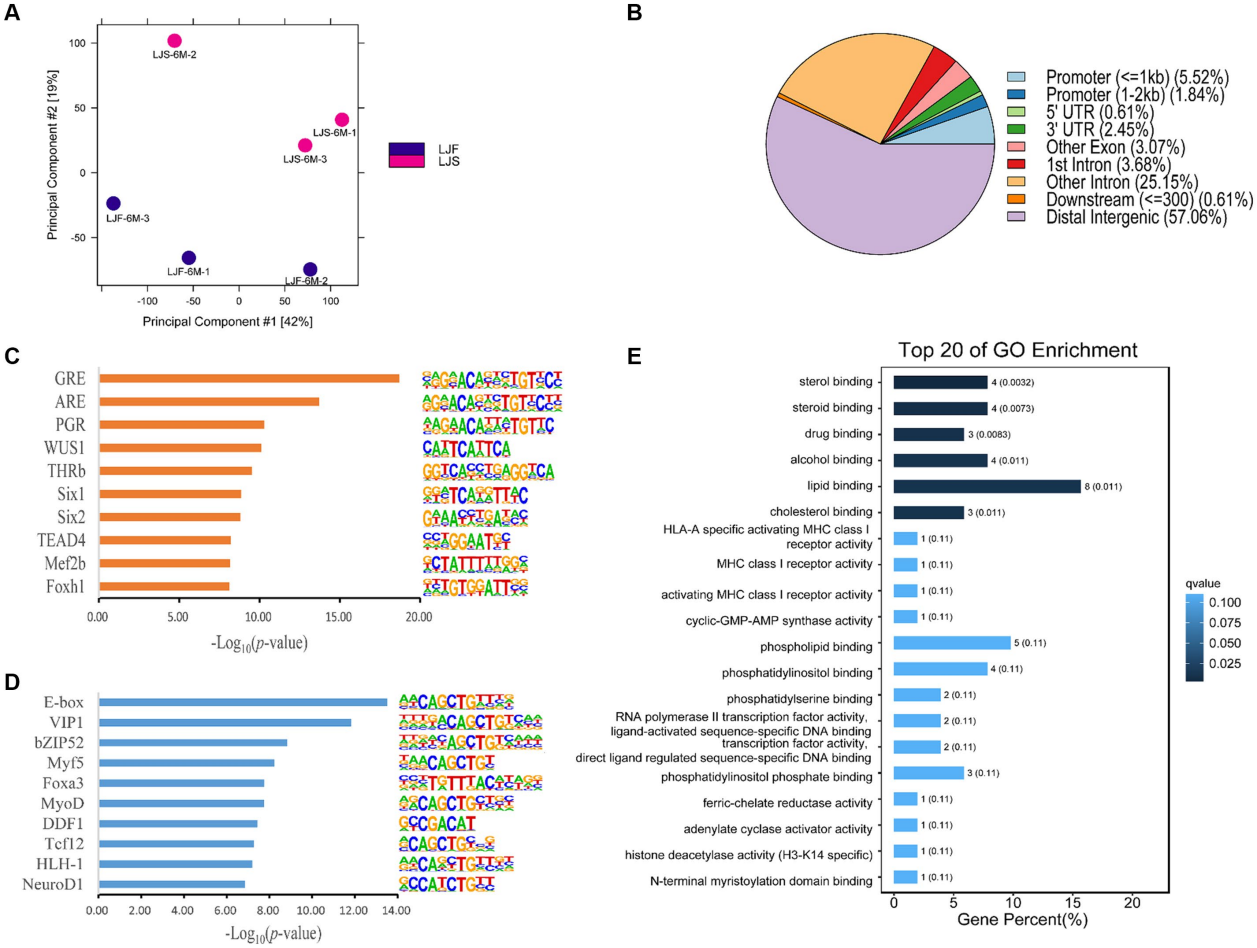
Muscle growth differences in Lijiang pigs revealed by ATAC-seq. **(A)** Principal component analysis of all features of ATAC-seq data for all samples. LJF, fast-growing Lijiang pigs; LJS, slow-growing Lijiang pigs. **(B)** Functional region distribution of differential OCRs. Identification of the top 10 enriched motifs in **(C)** upregulated peaks and **(D)** downregulated peaks from ATAC-seq data. **(E)** Gene Ontology (GO) enrichment analysis.

PCA was used to compare biological replicates and distinguish between the two lines of pigs based on growth rate, resulting in a clear separation (Figure 2A). These findings highlight significant differences between pigs with different phenotypes. Using DiffBind, 163 significantly differentially accessible regions (OCRs) were identified between the two phenotypes, with 110 OCRs showing upregulation and 53 OCRs exhibited downregulation. The differential OCRs primarily resided in distal intergenic and intronic regions (Figure 2B). Within these differential OCR regions, 59 motifs (*p* < 0.05) were predicted (Supplementary Table S4). Among the top 10 enriched motifs discovered in the upregulated OCRs, binding sites for Six1, Mef2b, Foxh1-known regulators of muscle fiber growth, were prominently present (Figure 2C). Moreover, the downregulated OCRs exhibited significant enrichment of motifs containing binding sites for Myf5, Foxa3, and MyoD, among others, and were associated with muscle growth (Figure 2D).

Analysis of differential gene expression in the OCRs revealed 126 genes with altered expression, including *PPARA*, *TNRC6B*, *NEDD1*, *FKBP5*, etc. (Supplementary Table S5). Subsequent GO and KEGG pathway analyses were conducted on these genes, revealing that the GO analysis primarily enriched for rhythmic processes, positive regulation of biological processes, metabolic processes, signal transduction, and developmental processes (Figure 2E). Furthermore, several KEGG pathways were implicated in the regulation of rhythmic processes, cellular differentiation processes, and lipid metabolism, including the circadian rhythm, circadian rhythm-ECM-receptor interaction, and aldosterone synthesis and secretion (Supplementary Figure S6).

### 3.3 Integration and analysis of highly genetically differentiated OCR

**Figure 3:**
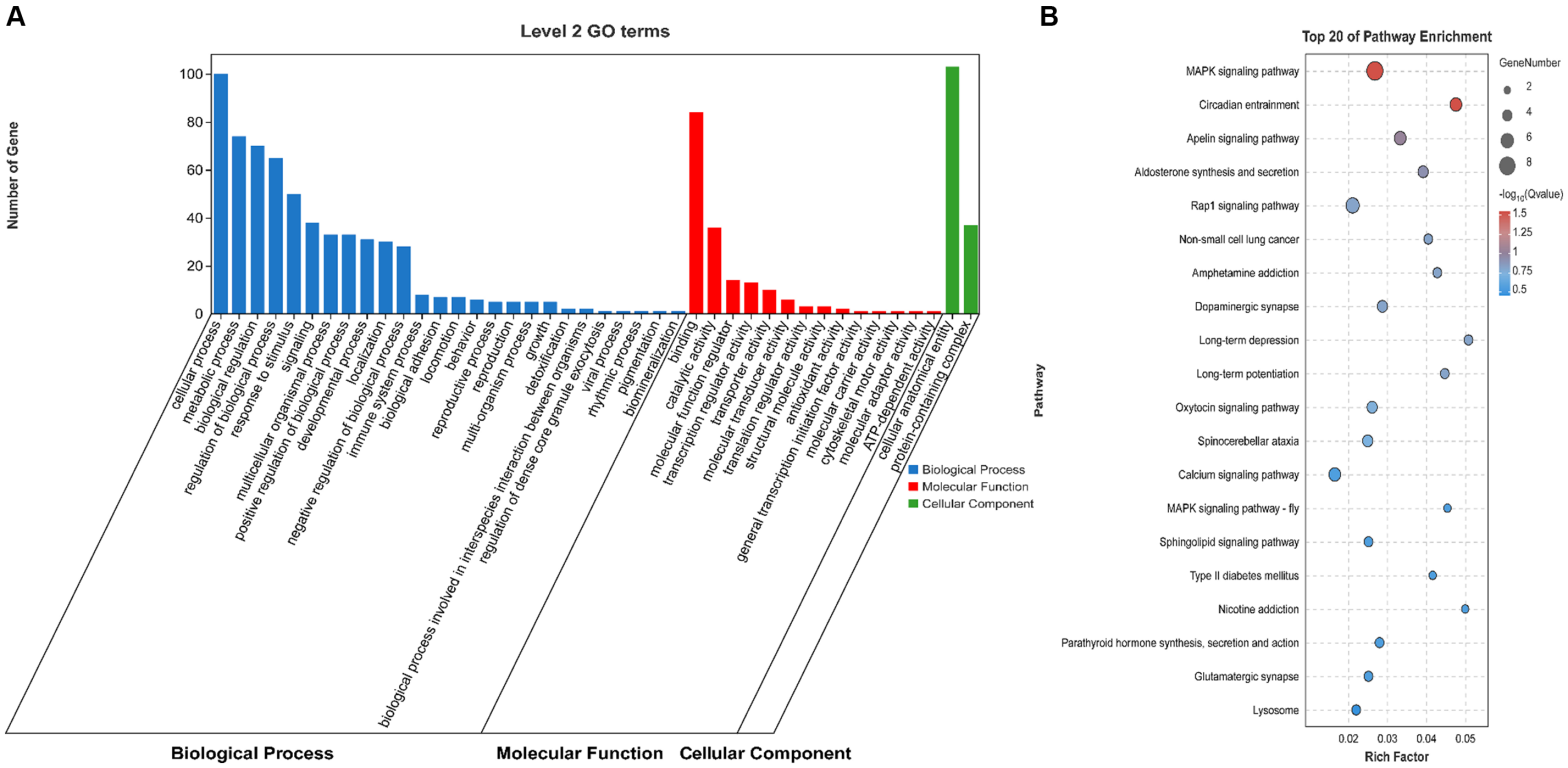
Genome analysis combined with ATAC-seq. **(A)** GO enrichment analysis histogram of highly genetically differentiated OCR genes. **(B)** Bubble map of Kyoto Encyclopedia of Genes and Genomes (KEGG) enrichment analysis of highly genetically differentiated OCR genes.

To explore the role of the OCRs in the genomic genetic differentiation region, 1,193 windows with significant genetic differentiation obtained from the overlapping genomic data and OCRs, and 140 genes, including *ARID2*, *CaCNA1H*, *JAK3*, and *PLPPR2*, were identified (Supplementary Table S5). *SAV1*, *SLC4A10*, *PRKCZ*, *ARID2*, and *SPTBN4* were enriched in the growth and development of muscle fibers based on GO terms (Figure 3A). *CACNA1H*, *CACNA1A*, *FGFR4*, and *PRKCG* were enriched in the MAPK and, calcium signaling pathways and other pathways related to muscle tissue (Figure 3B).

### 3.4 Integration of differential OCRs with transcriptional mapping analysis

**Figure 4:**
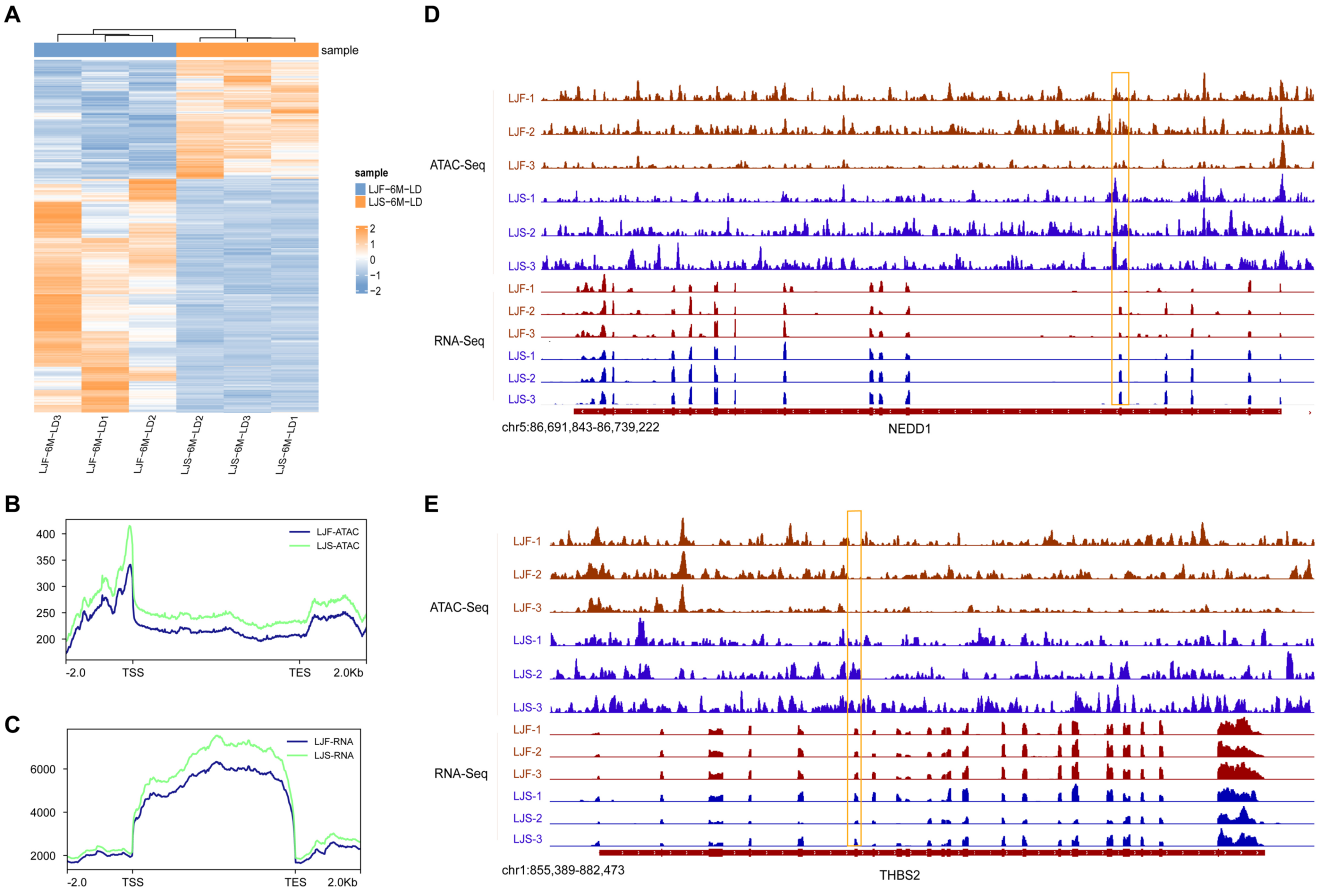
Overview of DEGs identified by ATAC-seq and RNA-seq. **(A)** Heatmap illustrating the differentially expressed genes (DEGs) identified through RNA-seq analysis. Signal density distribution maps based on ATAC-seq **(B)** and RNA-seq **(C)** data, with genomic regions mapped on the *x*-axis and signal densities on the *y*-axis. TSS, transcription start site; TES, transcription end site. Integrative Genomics Viewer snapshots showcasing the **(D)** *NEDD1* and **(E)** *THBS2* loci.

To explore the correlation between the openness of OCRs and gene expression, we integrated ATAC-seq and RNA-seq analyses. Initially, we identified all 508 genes with significant differences in the transcriptome data between LJS and LJF (Figure 4A; Supplementary Table S5). Subsequently, we analyzed the chromatin accessibility data around the TSS of genes identified from the ATAC-seq data. Signal density distribution plots showed that in both ATAC-seq and RNA-seq data, the LJS group had a higher signal density than the LJF group (Figures 4B,C). By applying |fold| > 1 and adjusted *p* < 0.05 as criteria for screening genes with differential expression in fast-growing and slow-growing individuals using transcriptome data, a combined total of eight common genes with differential expression were detected when combined with ATAC analysis, including *SCARB2*, *NEDD1*, *TMEM266*, *THBS2*, *HABP2*, *MYF6*, *FKBP5*, and ENSSSCG00000028777 (Figures 4D,E). Notably, *NEDD1* exhibited lower gene expression and chromatin openness levels in the LJF group compared to the LJS group; however, *THBS2* showed lower chromatin openness in the LJF group while having higher gene expression levels than the LJS group.

### 3.5 Combined analysis of transcription and protein maps

**Figure 5:**
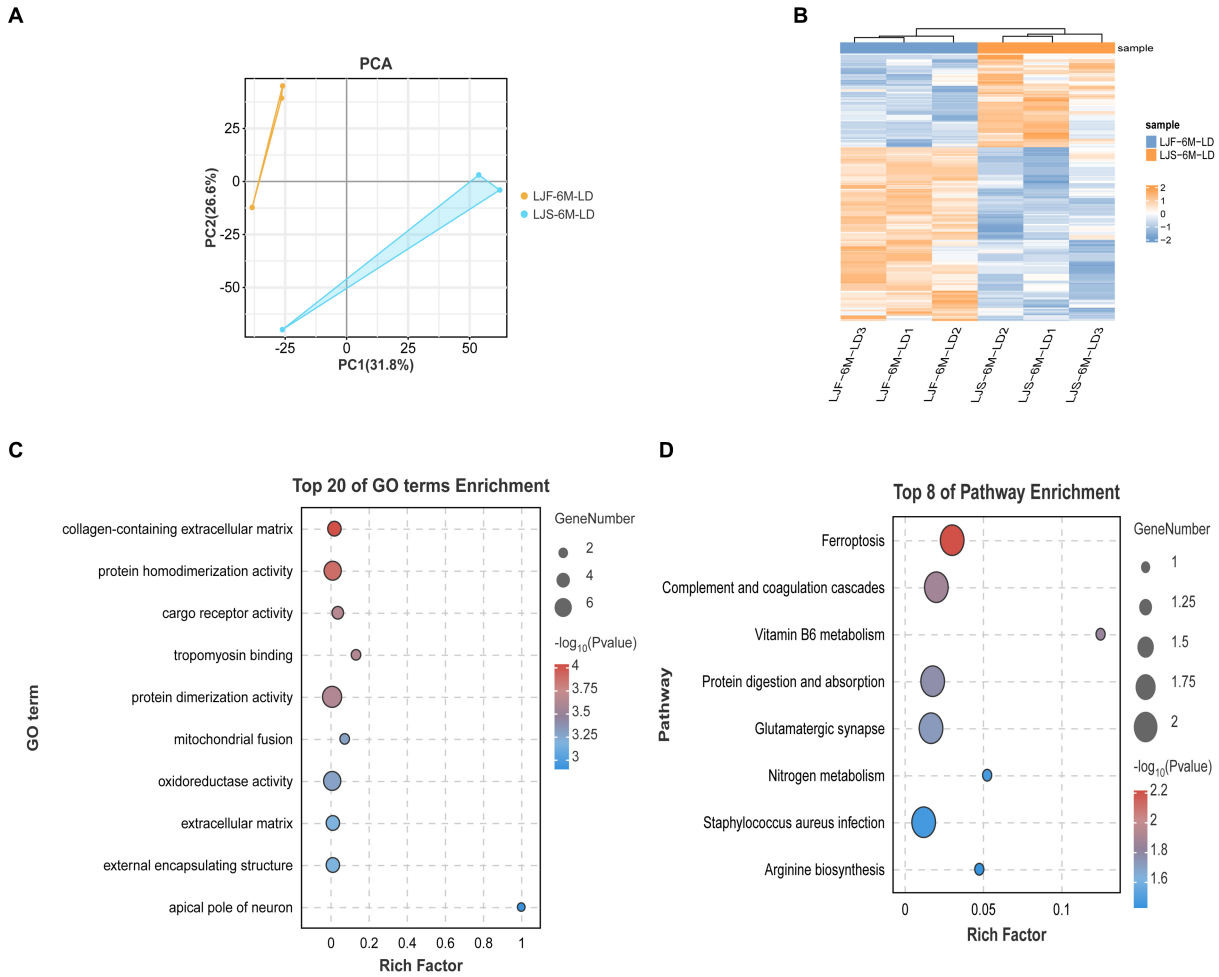
Combined analysis of protein map and DEGs. **(A)** Principal component analysis of all proteomic data. **(B)** Heat maps showing differential genes identified by proteome screening. **(C)** GO enrichment analysis of differential genes selected by combination of proteome and transcriptome. **(D)** KEGG enrichment analysis of differential genes selected based on combination of proteome and transcriptome data.

Principal component analysis showed that the proteome sequencing results of the two groups were significantly different and showed good reproducibility within groups (Figure 5A). A total of 454 differentially expressed proteins were identified (Figure 5B), including 26 that overlapped with DEGs, such as *FKBP5*, *HOMER2*, *COL14A1*, and *SCARB2* (Supplementary Table S5). These overlapping DEGs were enriched in GO terms, such as tropomyosin binding and mitochondrial fusion related to muscle development, as well as KEGG pathways, such as Arginine biosynthesis (Figures 5C,D). Notably, *FKBP5* and *SCARB2* were identified using combined ATAC-seq and transcriptome analysis.

### 3.6 Correlation and joint analysis of ATAC-seq, transcriptome, and proteome data

**Figure 6:**
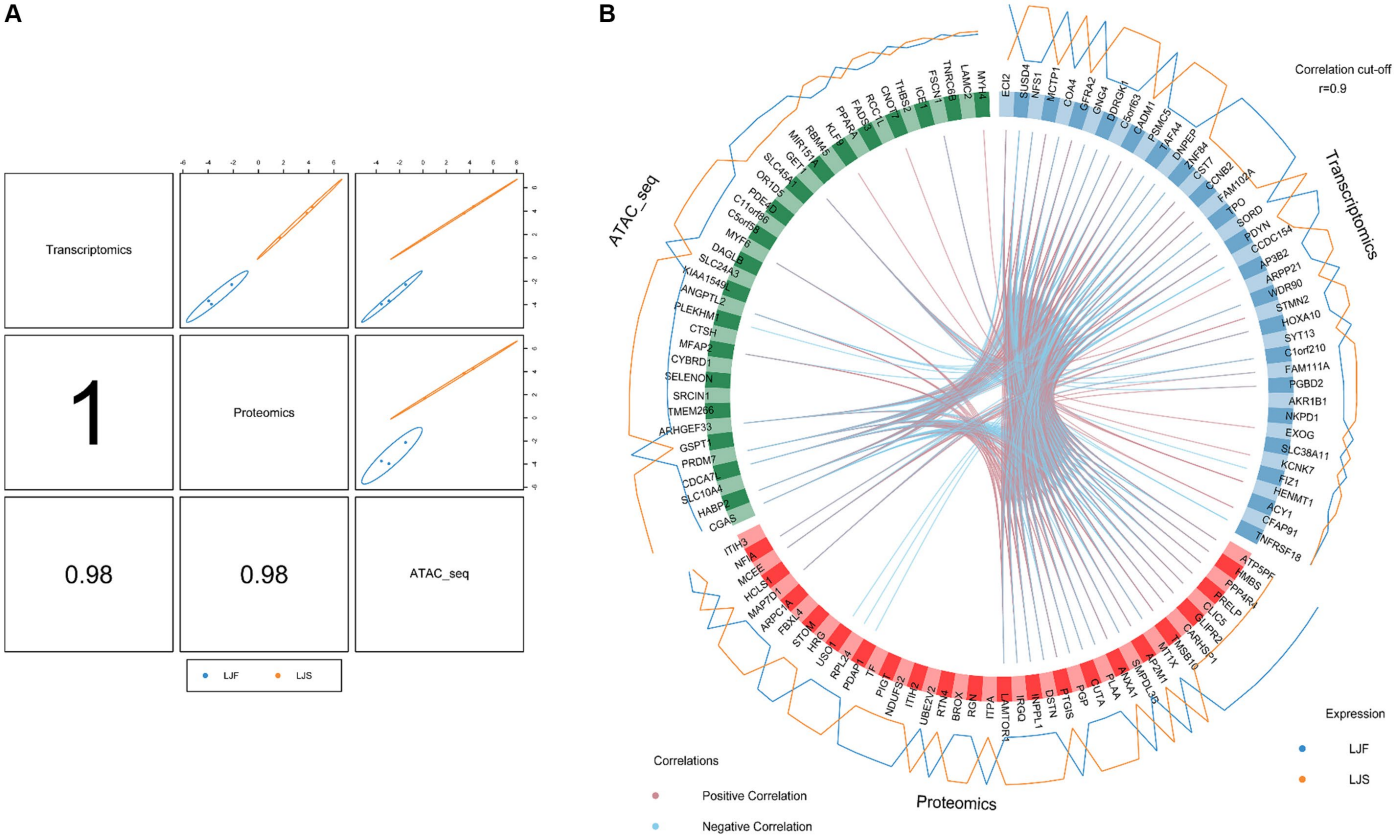
Multiomics analyses using ATAC-seq, transcriptome, and proteome datasets. **(A)** Correlations among first principal components of the respective identified features from ATAC-seq, transcriptome, and proteome datasets. **(B)** Correlations between genes identified using ATAC-seq, transcriptome, and proteome datasets, respectively. Genes categorized by dataset origin; inner circle lines denote inter-gene correlations; outer lines represent gene expression levels in LJF and LJS groups.

To joint multi-omics data and explore genetic differences between LJF and LJS Lijiang pigs, we combined ATAC-seq, transcriptome, and proteome datasets following differential analysis and gene set selection. Integration involved GO enrichment analysis, resulting in identification of 11 significantly enriched GO terms (adjusted *p* < 0.05), including glycogen metabolic process and skeletal muscle cell differentiation relevant to muscle growth (Table 1). Principal component analysis (PCA) of the three omics expression matrices of LJF pigs revealed strong correlations among datasets (Figure 6A). Genes exhibiting correlations greater than 0.9 across ATAC-seq, transcriptome, and proteome datasets were selectively retained within the circular plot (Figure 6B). Examples include *MYF6* and *HABP2* from ATAC-seq, *ECI2* and *SUSD4* from the transcriptome, and *ATP5PF* and *HMBS* from the proteome. These genes were identified based on their strong inter-omic correlations, highlighting potential regulatory or functional relationships across multiple molecular layers.

**Table 1:** The significantly enriched GO terms identified across ATAC-seq, transcriptome, and proteome datasets using ActivePathways.

ID	Term descriptions	Adjusted *p*-value
GO:0005977	Glycogen metabolic process	0.0076
GO:0005978	Glycogen biosynthetic process	0.0001
GO:0006511	Ubiquitin-dependent protein catabolic process	0.0443
GO:0010629	Negative regulation of gene expression	0.0014
GO:0030199	Collagen fibril organization	0.0013
GO:0032922	Circadian regulation of gene expression	9.0015 e-06
GO:0035914	Skeletal muscle cell differentiation	0.0045
GO:0043065	Positive regulation of apoptotic process	0.0210
GO:0043161	Proteasome-mediated ubiquitin-dependent protein catabolic process	0.0024
GO:0043588	Skin development	0.0198
GO:0051603	Proteolysis involved in protein catabolic process	0.0210

## 4 Discussion

For the first time, we investigated chromatin accessibility in Lijiang pigs which show significant variation in growth rates within the same breed. By integrating genomic genetic differentiation data, OCRs, transcriptome gene expression differences, and differentially expressed proteins, we identified the key genes and transcription factors associated with growth rate differences. This is despite focusing on a smaller dataset compared to similar analyses. A comparison of the two groups within the same breed ensured a direct link between DEGs and transcription factors and phenotypic variations in growth rate. This highlights their potential role in muscle growth mechanisms and, call for further exploration.

### 4.1 Disparities in muscle growth between fast-growing and slow-growing Lijiang pigs were examined through OCR analysis

A detailed comparison was performed to assess the differences in chromatin accessibility between the two groups. Notably, the upregulated OCRs in the LJS group exhibited significant enrichment in Six1 and other transcription factors, whereas Myf5 and MyoD were prominently enriched in the downregulated OCRs. Six1, Myf5 and MyoD are the key transcription factors involved in muscle fiber development. Mouse embryos lacking Six1 exhibit severe defects in muscle generation (34). MyoD promotes skeletal myogenesis by upregulating pre-myogenic mesodermal factors such as Six1 (35), and knockdown of Six1 leads to decreased expression levels of MyoD in myoblasts (36). Satellite cells activate MRF4 protein expression after multiple rounds of expansion, and the majority of cells fuse into myofibers that express MRF4, but have downregulated Myf5 levels. A minority of cells return to quiescence and do not express either gene (37). It is plausible that other transcription factors may upregulate Six1, whereas a negative feedback regulatory pathway inhibits adult pig myoblast proliferation, leading to a more quiescent state and downregulation of MyoD and Myf5 expression in the LJS group. During the later stages of fibroblast differentiation, Myc is highly expressed and suppresses fibroblast differentiation by repressing MyoD expression, thereby promoting muscle cell proliferation and hypertrophy. This finding supports our hypothesis (38, 39).

In addition, the researchers have also identified other key transcription factors involved in muscle fiber growth, including TEAD1, TEAD4, the Mef2 transcription factor family, Six2, JunB, Fos, Fosl2, Atf3, and MyoG. These have been demonstrated to affect myofiber growth. However, Foxh1, TCF12, and RBFox2 have not been shown to be related to myofiber growth. TEAD1 and TEAD4 are members of the TEA domain family, which is involved in muscle fiber-type conversion (40). Knockdown of TEAD4 affects the adipogenic differentiation of muscle-derived stem cells (41). The Mef2 family initiates the expression of genes related to muscle growth and differentiation, with Mef2a and Mef2c playing essential roles (42). Mice lacking Mef2a show impaired muscle regeneration (43), whereas the absence of Mef2c results in abnormal muscle development and neonatal death (44). Loss of Mef2a, Mef2c, and Mef2d prevents satellite cell differentiation (45). Additionally, Six2 overexpression enhances the expression of the proliferation markers PCNA and CCNB23, indicating that Six2 promotes cell proliferation (46). The interaction between c-Fos and c-Jun results in the formation of AP-1, a regulatory factor that plays a significant role in muscle cell proliferation (47). FosB and Fos are transcription factors belonging to the Fos subfamily, which is closely linked to the differentiation process of bovine satellite cells (48), and Fosl2, which cooperates with MyoD, also plays a role in regulating muscle growth and development. Fosl2 is a member of the AP-1 transcription factor family and is involved in glycogen regulation in chicken muscle (49). Hu et al. found that silencing Fosl2 led to the upregulation of MyoD, MyoG, Myh2, and Myh4, whereas silencing JunB resulted in increased expression levels of MyoD, Myh1, Myh2, and Myh4. JunB is a transcription factor that plays a pivotal role in the maintenance of muscle quality by inhibiting myostatin production. It prevent Foxo1 from binding to atrogin-3/MuRF promoters, thereby promoting muscle fiber hypertrophy and preventing muscle atrophy by preventing the binding of Foxo1 to the atrogin-3/MuRF promoter (50). These studies highlight the significant roles of Fosl2 and JunB as transcription factors in muscle growth and development (51). Atf3, another member of the AP-1 family, regulates H2B expression and influences satellite cell aging (52). TCF12, a member of the bHLH E-protein family, binds to the E-box regions of MyoD and MyoG, thereby promoting muscle cell proliferation and differentiation (37, 53). Foxh1 has been shown to promote the generation of induced pluripotent stem cells and regulate gene expression during reprogramming (54, 55). TCF12 deficiency leads to abnormal muscle development due to the dysregulation of genes related to muscle development and aberrant chromatin accessibility (56). Additionally, RBFox2 deficiency induces mitochondrial abnormalities in mature muscle cells in rats (57). It would be interesting to ascertain whether Foxh1, TCF12, and RBFox2 influence myofiber development in pigs.

### 4.2 Differences in chromatin accessibility for high genetic differentiation

By integrating genomic and ATAC-seq technologies, we identified *SAV1*, *CACNA1H*, *PRKCG*, *FGFR4*, *JAK3*, and other genes strongly associated with muscle fiber growth and development, that are highly genetically differentiated and localized in the open region of the chromatin, suggesting that they are likely to play important roles in regulating muscle development in Lijiang pigs and have the potential to be used as molecular markers to assist in Lijiang pig selection. Research has shown that reducing *SAV1* expression levels inhibits the Hippo pathway, thereby enhancing the proliferation of skeletal muscle satellite cells (58). The mutations p.V681L and p.D1233H in *CACNA1H* lead to congenital muscular atrophy (59), and the down-regulation of *CACNA1H* promotes muscular tube atrophy of skeletal muscle, which may be related to Ca^+^ disorders (60). *PRKCG*, also known as PKC-γ, blocks sustained muscle contraction induced by epidermal growth factor (61). *FGFR4* promotes progenitor cell differentiation in chicken embryonic muscle (62) and *MyoD* promotes muscle regeneration by inducing *FGFR4* transcription through the binding and activation of TEAD2 (63). *JAK3* regulates skeletal muscle growth and development through the JAK–STAT signaling pathway, down-regulates *STAT1* expression, and up-regulates *STAT3* expression to promote precocious myogenic differentiation (64).

### 4.3 Integration of chromatin accessibility disparities with gene transcription and expression variations in the skeletal muscles of fast-growing and slow-growing Lijiang pigs

The integration of ATAC-seq and RNA-seq data revealed 11 common DEGs between the LJF and LJS groups. Among these genes, *NEDD1*, *THBS2*, and *FKBP5* are closely associated with muscle fiber development. Previous studies have shown that *NEDD1* is essential for mitosis by regulating the localization of the γ-tubulin complex at centrosomes, ensuring proper microtubule nucleation and spindle assembly for normal cell growth and development (65–67). The ectopic expression of *NEDD1* can lead to growth inhibition (68), which is consistent with the findings of transcription factor studies. Differential expression of *NEDD1* may lead to differences in growth and development among different types of Lijiang pigs. *THBS2*, a marker of late tendon development in mice, has not yet been studied for its effects on porcine muscle development (69, 70). In the present study, we observed that the chromatin openness of *THBS2* in the LJS group exhibited higher levels compared to those in the LJF group. However, the gene expression was lower in the LJS group than in the LJF group, which may be attributed to the presence of transcription inhibitors.

*FKBP5* and *SCARB2* were identified as common differentially expressed genes in ATAC-seq, transcriptome, and proteome analyses comparing fast and slow-growing Lijiang pigs. KLF15 promotes myoblast differentiation by binding to the promoter region of *FKBP5* and activating its expression of *FKBP5* (71). *FKBP5* affects the differentiation of myoblast cells through two mechanisms: preventing the formation of the main inhibitor of differentiation, the cyclin D1-Cdk4 complex, by sequestering Cdk4 in the Hsp90 storage complex, and promoting Cdk isomerization to inhibit the phosphorylation of Thr172, thus activating Cdk4 (72). In Muscovy ducks, three SNPs of *FKBP5* were found to be significantly correlated with body weight traits (73). This indicates that *FKBP5* is an important gene that affects the growth and development in muscle fibers, which is a potential molecular marker for muscle development traits of Lijiang pigs. Pathogenic variants of the *SCARB2* gene have been linked to symptoms such as muscle spasm and ataxia in patients (74); however, the regulatory mechanism of *SCARB2* in skeletal muscle growth and development deserves further verification.

Joint analysis of ATAC-seq, transcriptome, and proteome datasets enabled the identification of significantly enriched pathways and co-regulated genes, suggesting potential regulatory networks involved in transcriptional, post-transcriptional, and translational processes in LJF and LJS pigs. For instance, *MYF6*, a member of the *MyoD* gene family, plays a pivotal role in multiple stages of muscle fiber development. It is regulated by various ncRNAs, which influence the maturation and differentiation of porcine myotubes (75, 76).

In conclusion, in this study, we identified several genes and transcription factors using ATAC-seq-based multi-omics. These can be further explored and validated to ascertain their effects on porcine muscle development. However, due to the unique characteristics of genomic resequencing data, additional experiments are required to pinpoint critical loci. Therefore, genomic resequencing data were not included in the final multi-omics integration. Additionally, three full-siblings of fast-growing Lijiang pigs and three slow-growing Lijiang pigs were selected for the experiments in this study, and the effect of family lineage effects on the results was not excluded. Future research should focus on exploring more sophisticated and comprehensive methods for multi-omics integration.

## 5 Conclusion

In this study, we conducted an extensive analysis of chromatin accessibility, transcription factor prediction, gene transcription, and differential protein expression during muscle growth in pigs with different growth rates. ATAC-seq technology was used to identify transcription factors such as Six1, Mef2b, Myf5, MyoD, Foxh1, TEAD1, and TEAD4. By integrating various OCRs with genomic variations, genes such as *SAV1*, *CACNA1H*, *PRKCG*, and *FGFR4* were found to as have significant differences in the OCRs. We also examined the expression and transcriptional variances of *NEDD1*, *THBS2*, *MYF6*, *SCARB2*, and *FKBP5* by integrating ATAC-seq data DEGs identified in the transcriptome. Notably, *FKBP5* and *SCARB2* genes, which showed distinctions in OCRs, transcription levels, and protein levels between fast and slow-growing Lijiang pigs, were identified. Based on the joint analysis of ATAC-seq, transcriptome, and proteome datasets, we identified significant enrichment of processes related to glycogen metabolism and skeletal muscle cell differentiation. Furthermore, through intergroup correlation analysis, key genes such as *MYF6* and *HABP2* were identified. The identified candidate genes and transcription factors are crucial for regulating pig muscle growth, highlighting the need for further investigation. The findings of this study offer valuable perspectives for future investigations on breed-specific variations in growth rate, muscle development, and multi-omics correlation analyses.

## Data Availability

The ATAC-seq datasets presented in this study are available in the NCBI database under accession number PRJNA1094405. Additional omics data and their corresponding accession numbers are provided in the article.
