# Supplementary figures and images for: Muscle growth differences in Lijiang pigs revealed by ATAC-seq multi-omics
Source: Front Vet Sci. 2024 Aug 26;11:1431248. doi: 10.3389/fvets.2024.1431248 (PMC11381499; doi:10.3389/fvets.2024.1431248)

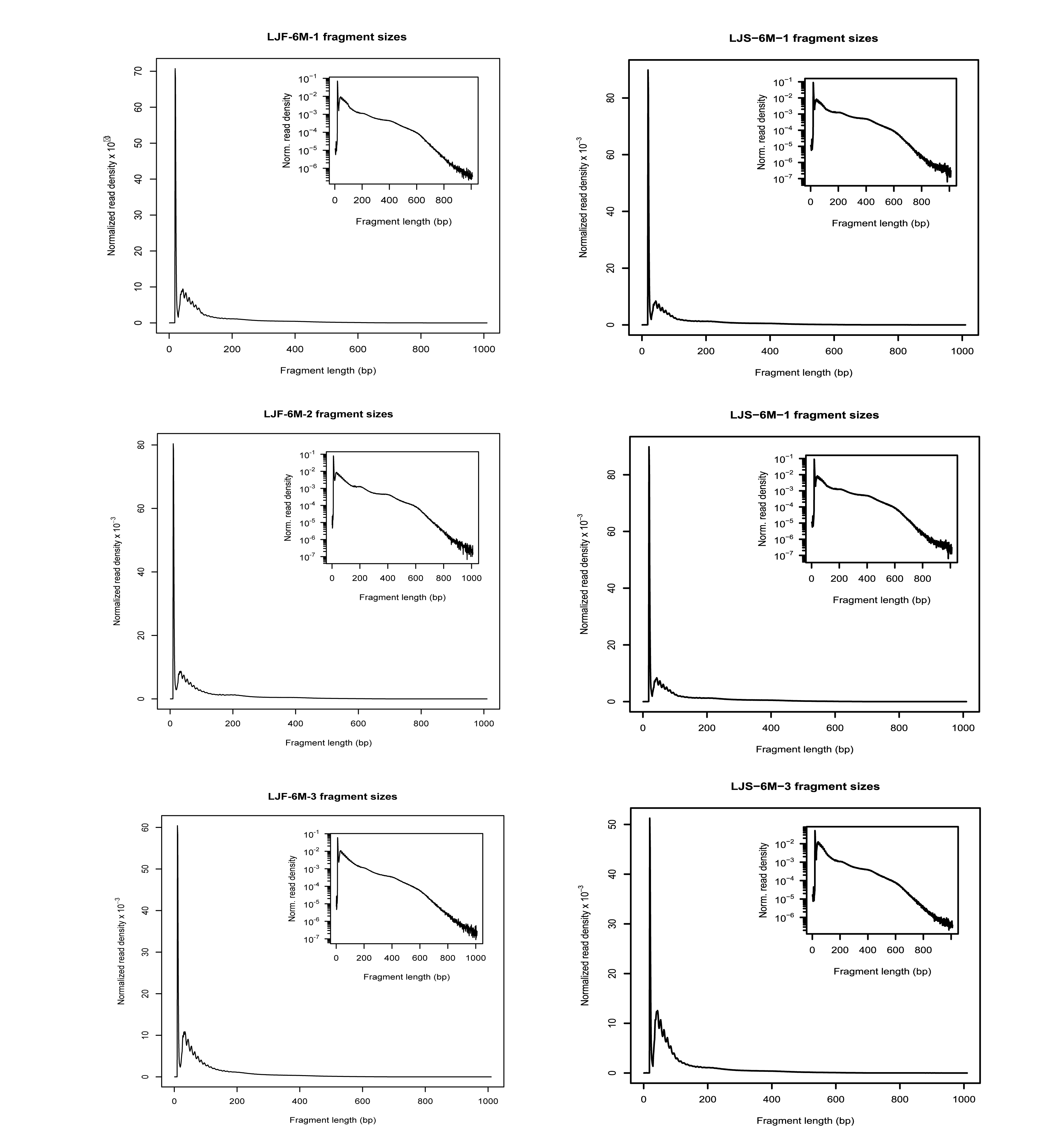

Supplement: Supplementary file 1 [file Data_Sheet_1.zip › Supplementary/S3.jpg]

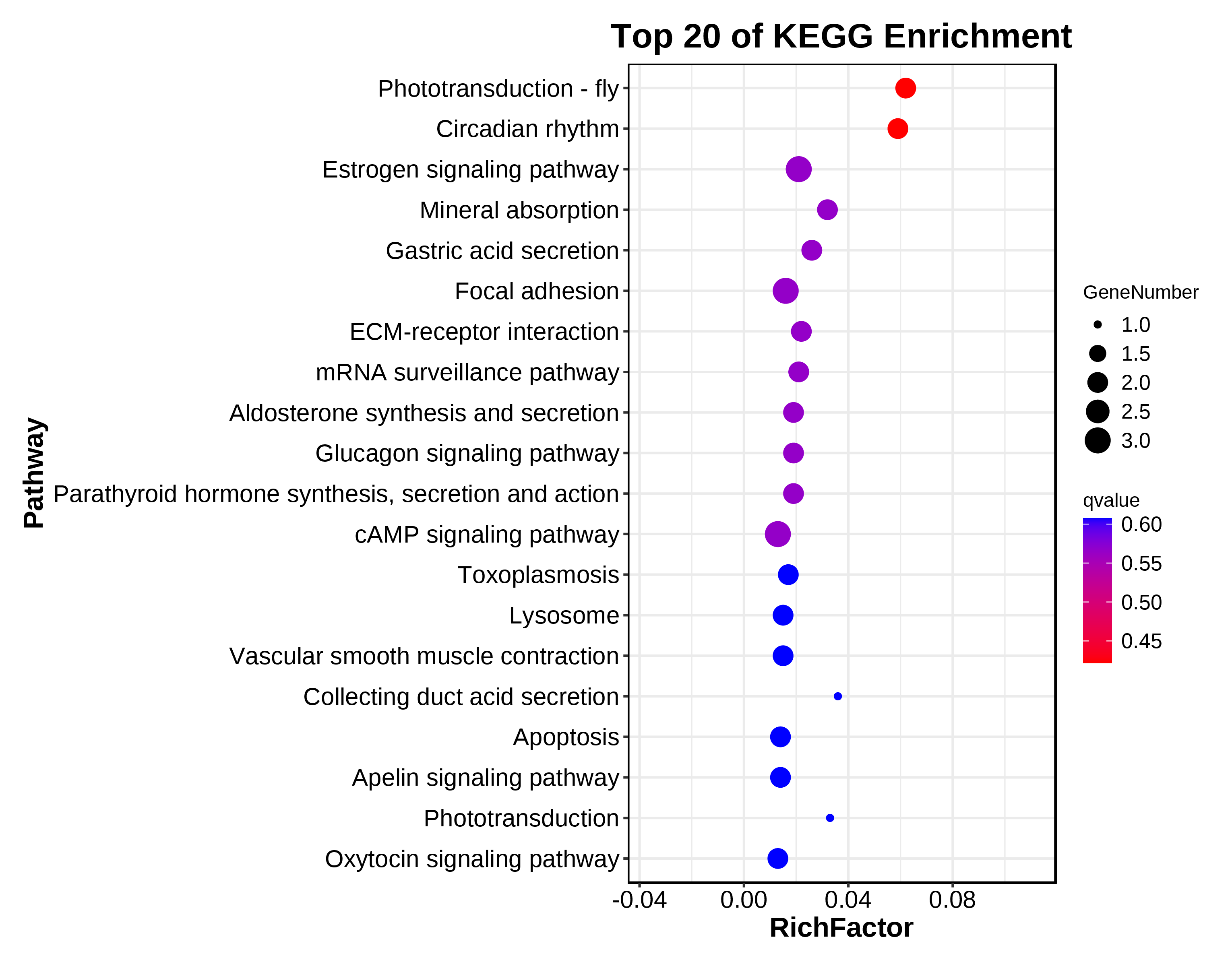

Supplement: Supplementary file 1 [file Data_Sheet_1.zip › Supplementary/S6.png]
